# Endothelial calcium dynamics elicited by ATP release from red blood cells

**DOI:** 10.1038/s41598-024-63306-2

**Published:** 2024-06-12

**Authors:** Ananta Kumar Nayak, Sovan Lal Das, Chaouqi Misbah

**Affiliations:** 1 CNRS, LIPhy, Université Grenoble Alpes, 38000 Grenoble, France; 2 Physical and Chemical Biology Laboratory, and Department of Mechanical Engineering, Indian Institute of Technology Palakkad, Palakkad, 678623 India

**Keywords:** Biophysics, Mathematics and computing, Physics

## Abstract

Red blood cells (RBCs) exhibit an interesting response to hydrodynamic flow, releasing adenosine triphosphate (ATP). Subsequently, these liberated ATP molecules initiate a crucial interaction with endothelial cells (ECs), thereby setting off a cascade involving the release of calcium ions (Ca$^{2+}$). Ca$^{2+}$ exerts control over a plethora of cellular functions, and acts as a mediator for dilation and contraction of blood vessel walls. This study focuses on the relationship between RBC dynamics and Ca$^{2+}$ dynamics, based on numerical simulations under Poiseuille flow within a linear two-dimensional channel. It is found that the concentration of ATP depends upon a variety of factors, including RBC density, channel width, and the vigor of the flow. The results of our investigation reveals several features. Firstly, the peak amplitude of Ca$^{2+}$ per EC escalates in direct proportion to the augmentation of RBC concentration. Secondly, increasing the flow strength induces a reduction in the time taken to reach the peak of Ca$^{2+}$ concentration, under the condition of a constant channel width. Additionally, when flow strength remains constant, an increase in channel width corresponds to an elevation in calcium peak amplitude, coupled with a decrease in peak time. This implies that Ca$^{2+}$ signals should transition from relatively unconstrained channels to more confined pathways within real vascular networks. This notion gains support from our examination of calcium propagation in a linear channel. In this scenario, the localized Ca$^{2+}$ release initiates a propagating wave that gradually encompasses the entire channel. Notably, our computed propagation speed agrees with observations.

## Introduction

Red blood cells (RBCs) are commonly known to transport oxygen to the tissues and to carry metabolic wastes from the tissues^1^. However, RBC also transport and release an important signaling molecule, adenosine triphosphate (ATP)^2–4^. ATP is an important second messenger, which helps in transmitting information from the surrounding of endothelial cell (EC) into cytoplasm of the EC. This is only possible by activating a cascade of reactions on plasma membrane (PM) of the EC^5,6^. A basic consequence of ATP is the release of sequestered Ca$^{2+}$ from the endoplasmic reticulum (ER) of EC into the cytoplasm^7^.

The endothelium, a confluent monolayer of ECs, forms the inner side of all vessel walls, so that it is constantly come in contact with the bloodstream. A basic function of the EC is to selectively allow the transportation of oxygen, nutrients, and ions to the tissue for a smooth functioning of organs. In addition, ECs synthesize one of the important vasodilators, namely the Nitric Oxide (NO), from the precursor L-arginine^8,9^. The production of NO is controlled by the endothelial nitric oxide synthase (eNOS) enzyme. However, the activation eNOS is regulated by the cytoplasmic Ca$^{2+}$ concentration^4,10–12^. Thus, Ca$^{2+}$ is treated as an important ion and a second messenger that controls many cellular functions at different time scales^13^. Ca$^{2+}$ is also well known to regulate several upstream and downstream pathways such as PLC-dependent IP$_3$ production due to increase in the cytoplasmic Ca$^{2+}$ concentration^14^ and calmodulin-eNOS mediated NO synthesis^15^, respectively. Overall, Ca$^{2+}$ implicitly controls many cellular functions such as gene transcription, fertilization, and vasodilation^13^.

NO molecules produced due to the activation of eNOS by Ca$^{2+}$ rapidly diffuse into the neighboring ECs, the bloodstream, and the vascular smooth muscle cells (SMCs). This leads to the relaxation of SMCs^16^, reducing thereby the vascular resistance to blood flow by dilating the diameter of the vascular wall locally. In other words, NO helps in maintaining vascular tone within the vascular system^17^. In addition, NO in the bloodstream ensures several other functions such as prevention of the adherence and aggregation of platelets to the EC, and the adhesion of leukocyte and infiltration, as well as the proliferation of SMCs. Furthermore, any imbalance in NO production leads to disruption of vascular homeostasis. As a result, several life-threatening vascular diseases such as aortic aneurysms, hypertension, and atherosclerosis may arise^17,18^. An essential first step is to elucidate the early process leading to the rise of Ca$^{2+}$ in the cytoplasm due to ATP released from RBCs in the vascular vessels. This is the first objective of this paper.

The role of ATP in the rise of cytoplasmic Ca$^{2+}$ concentration is quite well understood. This is due to the activation of purinergic receptors (P2Y$_2$) by ATP^5,6^. For example, binding of ATP to P2Y$_2$ receptors on the surface of ECs leads to inception of a second messenger called inositol trisphosphate (IP$_3$), which eventually helps in mobilization of sequestrated Ca$^{2+}$ from ER^19^. The main source of ATP in the bloodstream originates from RBCs^2^. These ATP molecules help in mobilization of ER Ca$^{2+}$. However, another source of ATP originates from the ECs, directly due to shear stress, triggering thus Ca$^{2+}$. ATP (from RBC and EC) activates G-protein (which is linked to purinergic receptors) leading to the production IP$_3$ molecules and then to the release of Ca$^{2+}$ from the ER^20^.

The typical ATP concentration inside RBC is in the range of mM^21^, whereas it is in the range of $\mu$M in the plasma^22^. This means that ATP released from RBCs may potentially exceed the need of ATP in the plasma, and this may become physiologically harmful^23^. Therefore, ATP concentrations in the bloodstream must be maintained within the acceptable physiological range in the plasma (i.e., of the order of $\mu$M). This regulation is achieved thanks to an enzyme, ectonucleotidase, present on the surface of the EC, which hydrolyzes ATP into adenosine diphosphate (ADP) and eventually, ADP to adenosine monophosphate (AMP) and AMP to adenosine (AD). This means that there is a need to take into account the role of the EC in maintaining the physiological level of ATP in the plasma. Thus, we will consider here ATP release from both RBCs (the major source) and EC, as well as hydrolysis of ATP by the EC.

The elucidation of ATP pathways in the release by RBCs has improved thanks to in-vitro experiments^2^. It has been reported that ATP released from RBCs follows two pathways: (1) the activation of Pannexin-1 (Px$_1$) hemichannels due to mechanical stress experienced by the membrane of RBC in flow and (2) the stretching of RBC membrane (due to topological defects in the spectrin-actin network) leads to activation of the cystic fibrosis transmembrane conductance regulator (CFTR), which ultimately up-regulates the Px$_1$ hemichannels. Zhang et al.^24^ have recently proposed a phenomenological model based on the above experimental results^2^. Their model accounted for the above two pathways and agreed with the experimental observations^2^. However, the experimental results of Forsyth et al.^2^ and the numerical study of Zhang et al.^24^ were performed for a very dilute suspension of RBCs under a shear flow. The realistic blood flow situation in circulatory system is governed by the Poiseuille flow and the complex hydrodynamic interactions among RBCs, where the total volume of RBCs in a healthy situation may reach up to 45 $\%$ (designated as Hematocrit, *Hct*). Gou et al.^25^ extended the study of Zhang et. al^24^ to the Poiseuille flow in a two-dimensional (2-D) straight channel for different flow conditions as well as for different RBCs hematocrits. However, the role of endothelium (i.e., ATP release from ECs, hydrolysis of ATP by the ectonucleotidase enzymes, and the activation of Ca$^{2+}$ signaling), were not studied. As a result, ATP concentrations in the channel increased with time without bound. Clearly, the role of the ECs must be incorporated into the model in order to maintain a stable ATP concentration. This is another goal of this study. Once this question is elucidated, we will be in a position to address the question of Ca$^{2+}$ signaling in the endothelium.

Our next goal will be the analysis of calciulm signaling due to ATP. In order to understand how the distribution of ATP on the surface of the endothelium affects the Ca$^{2+}$ signaling in the endothelium, Plank et al.^26,27^ proposed a model which couples the main three variables, namely IP$_3$ ($IP_3$), the cytoplasmic Ca$^{2+}$ ($Ca_c$), and the ER Ca$^{2+}$ ($Ca_s$) with a given distributions of ATP on the endothelium surface. Although they considered the role of endothelium on the steady concentrations of ATP distribution in the 2-D channel, the source of ATP was not known *apriori*. In addition, the Ca$^{2+}$ dynamics model used in these studies did not satisfy intracellular homeostasis. The intracellular homeostasis means that the concentrations of three important variables such as $IP_3$, $Ca_c$, and $Ca_s$ must return to their initial concentrations in the presence of ATP. To fill this gap, we have recently developed a minimal model in which the intracellular homeostasis was achieved thanks to the phosphorylation of P2Y$_2$ receptors upon binding of ATP molecules^28^.

In this study, we will treat RBCs under flow as the first main source of ATP. This source will be shown to depend on the spatial organization of RBCs, which is affected by the channel width, the hematocrit and the flow strength. In addition, we will take into account the role of ECs (i.e., ATP release from ECs, hydrolysis of ATP by the ectonucleotidase enzymes, and the activation of Ca$^{2+}$ signaling), which is an essential ingredient in order to achieve a steady pattern of ATP. Finally, we will use our recently developed model^28^ for Ca$^{2+}$ signaling, which takes into account the phosphorylation of P2Y$_2$ receptors upon binding of ATP molecules. This ingredient will be shown to ensure homeostasis for the three variables entering dynamics ($IP_3$, $Ca_c$, and $Ca_s$).

We have studied Ca$^{2+}$ signaling as a function of hematocrit, channel size, and flow strength. Additionally, we have analyzed the possibility for Ca$^{2+}$ propagation along the endothelium and determined the propagation speed. Our investigation reveals key findings: (1) The peak amplitude of Ca$^{2+}$ in EC increases proportionally with RBC concentration. (2) Higher flow strength increases the magnitude of Ca$^{2+}$ signals and reduces the time to reach the Ca$^{2+}$ peak, for a given channel width. (3) Increasing channel width, while flow strength remains constant, elevates Ca$^{2+}$ peak amplitude and reduces peak time. This suggests that Ca$^{2+}$ signals have the potential to propagate from a region of higher Ca$^{2+}$ (wide vessels) concentration to a region of lower Ca$^{2+}$ (small vessel diamaters) concentration within vascular networks at a speed of 5 $\mu$m/s. This study will offer a general basis for Ca$^{2+}$ signaling in the presence of blood flow along ECs, which can serve a general basis for a more elaborate analysis in complex vascular networks.

## Methods

**Figure 1:**
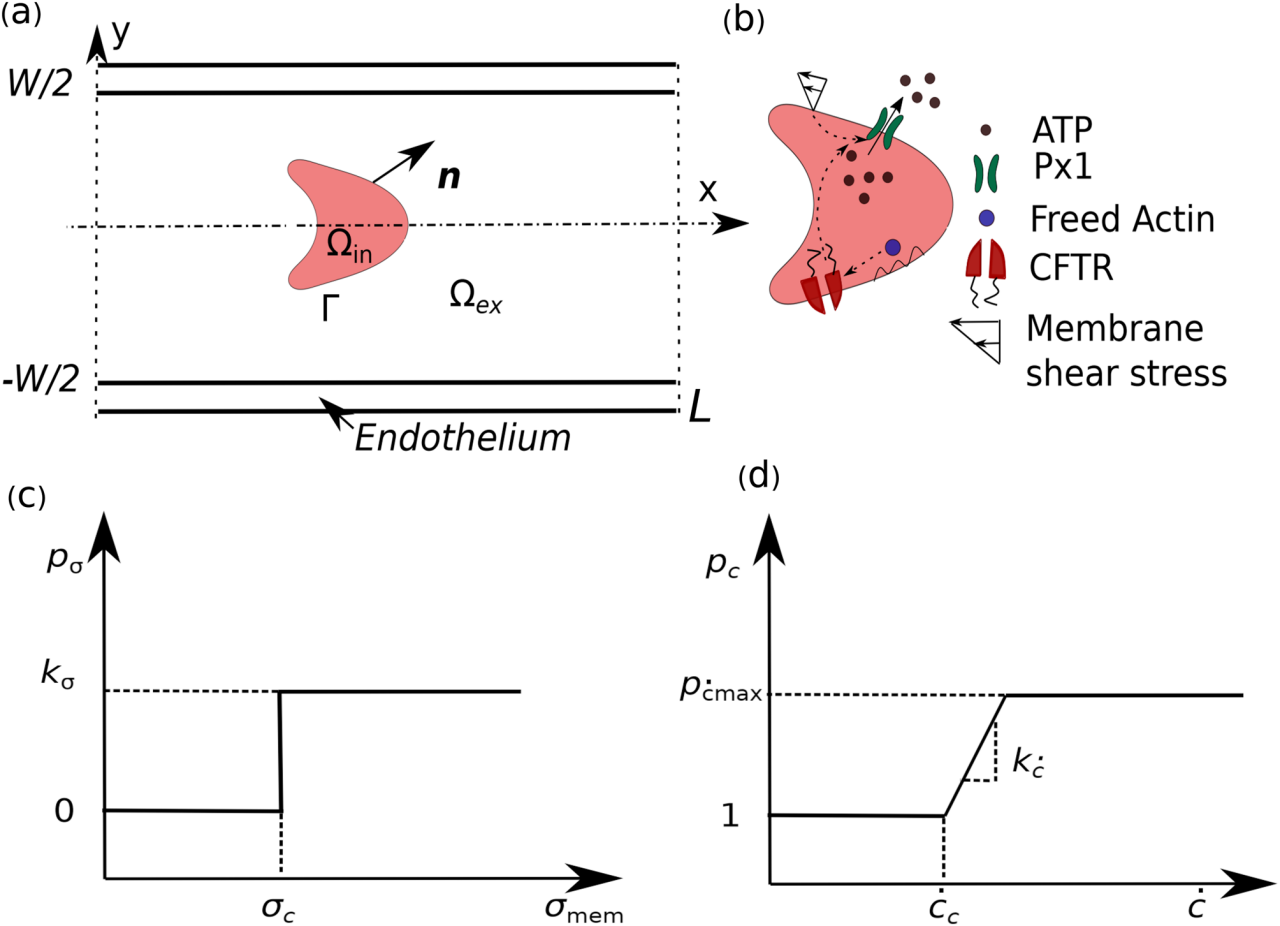
Schematic shows: (**a**) RBC is moving in a straight two-dimensional channel (2-D) which is lined with a layer of endothelial cells known as endothelium; (**b**) the pathways by which RBC releases ATP molecules when it is subjected to shear stresses; (**c**) the activation of pannexin-1 (Px$_1$) hemichannels when the membrane shear stress exceeds the critical shear stress; and (**d**) up-regulation of ATP release by the cystic fibrosis transmembrane conductance regulator (CFTR) through Px$_1$ when the curvature change rate exceeds the critical curvature change rate.

We consider the 2-D model for the sake of numerical efficiency, as we have already shown that the 2-D model captures the essential features of the three-dimensional (3-D) RBC model (such as shapes under flow^29^, cross-streamline noninertial migration velocity^30,31^, inclination angle of cell with respect to flow direction^32,33^, multilobe shapes^34^, and ATP release under flow^25^). Note that for ATP release, a topic of direct interest here, an even semi-quantitative agreement between 2D and 3D is obtained^25^. Due to the 2-D assumption, the cell boundary is one-dimensional (1-D) incompressible boundary and can only undergo bending (like for pure phospholipid membranes). The fluid flow inside and outside the RBC is described by the Navier-Stokes equations for an incompressible flow

1 $$\begin{aligned} \nabla \cdot {\textbf {v}} = 0, \end{aligned}$$

2 $$\begin{aligned} \rho \left( \frac{\partial {\textbf {v}}}{\partial t}+ {\textbf {v}} \cdot \nabla {\textbf {v}}\right) = -\nabla p + \nabla \cdot (\mu \nabla {\textbf {v}}) + {\textbf {F}}, \end{aligned}$$

where $\rho$ is the density of the incompressible fluid, *p* is the pressure, and $\mu$ is the dynamic viscosity of the fluid. In order to distinguish the dynamic viscosities of fluids in the interior and exterior of RBCs, we define a dimensionless number called the viscosity contrast, $\lambda$. $\lambda$ is defined as the ratio between the dynamic viscosity of the interior, $\mu _{\text {in}}$ to that of the exterior, $\mu _{\text {ex}}$ fluids of the RBC. In this numerical study, we use the physiological value of the viscosity contrast and the dynamics viscosity of the fluid present inside the RBC *i.e.,* $\lambda = 6$ and $\mu _{\text {in}} = 0.012$ Pa s^2^. The force, ${\textbf {F}}$ (N/m$^3$) in Eq. (2) is obtained from the deformation of the membrane due to flow. This force is distributed over fluids close to the RBC membrane using the immersed boundary method (IBM)^35^

3 $$\begin{aligned} {\textbf {F}} = \oint \limits _{\Gamma }\, {\textbf {f}}(s) \delta ({\textbf {x}}- {\textbf {X}}(s))ds \,, \end{aligned}$$

where ${\textbf {x}}\in [0,L] \times [-W/2, W/2]$ (*L* and *W* are the length and width of the channel, respectively), $\Gamma$ represents the integration along the contour of a RBC (shown in Fig. 1, and ${\textbf {X}}(s)$ is a set of Lagrangian points on the membrane in the x-y plane, *s* is being the local arc length.

This membrane force, ${\textbf {f}}(s)$ is obtained by the functional derivative of the Helfrich bending energy for the vesicle model. The Helfrich bending energy per unit length (J/m), *E* is defined as^36^

4 $$\begin{aligned} E({\textbf {X}}(s)) = \frac{\kappa }{2}\oint \limits _{\Gamma }\, c^2 ds + \oint \limits _{\Gamma }\, \zeta ds\,, \end{aligned}$$

where $\kappa$ is the bending modulus of the membrane ($\kappa = 3\times 10^{-19}$ J)^37^, *c* is the local mean curvature of the membrane, and $\zeta$ is the Lagrangian multiplier, which preserves the constant membrane perimeter, *P*. The membrane force, ${\textbf {f}}(s)$ (N/m$^2$) is obtained after taking the functional derivative of Eq. (4)^30^ and is written as

5 $$\begin{aligned} {\textbf {f}}(s) = \frac{\delta E}{\delta {\textbf {X}}(s)} = \kappa \left( \frac{\partial ^2 c}{\partial s^2} + \frac{c^3}{2}\right) {\textbf {n}} -\zeta c {\textbf {n}} + \frac{\partial \zeta }{\partial s} {\textbf {t}} \,, \end{aligned}$$

where ${\textbf {n}}$ and ${\textbf {t}}$ are the local unit outward normal and tangent vectors on the contour, respectively. The velocity of the Lagrangian points is obtained by interpolating the velocity of neighboring fluid nodes to the membrane using the IBM^35^, as follows

6 $$\begin{aligned} \frac{\partial {\textbf {X}}}{\partial t} = {\textbf {V}}(s) = \int \limits _{y}\, \int \limits _{x}\, \delta ({\textbf {x}} - {\textbf {X}}(s)) \textbf{v}({\textbf {x}}) dx dy\,. \end{aligned}$$

Subsequently, the position of the Lagrangian points of the membrane is updated using Euler method. We define a dimensionless number called the reduced area, $\nu$, which is defined as the ratio between the vesicle area, *A* and area of a circle having the same perimeter (*P*)*i.e.,*

$$\nu =\frac{4\pi A}{P^2}$$

where $A= \pi R_0^2$, $R_0$ is the characteristics radius of the RBC. In numerical simulation, we have taken, $\nu = 0.65$, which is the physiological value for a healthy RBC. We impose a pressure-driven Poiseuille flow, with a velocity field along the $x-$direction given by

$$v_x = v_{\text {max}}\left[ 1-\left( \frac{y}{W/2}\right) ^2 \right] .$$

where $v_{\text {max}}$ is the maximum flow velocity at the center of the channel. We assume no slip boundary conditions for the channel walls. The immersed boundary lattice Boltzmann method (IBLBM)^38^ is employed to numerically solve the Eqs. (1)-(6) for the fluid velocity field.

Once the velocity field is obtained in the channel, it is used to find the distribution of ATP in the channel. The ATP released from RBCs due to shear stress and cell deformation, diffuses in the suspending fluid and is advected by the flow. Thus, the advection–diffusion equation for ATP is

7 $$\begin{aligned} \frac{\partial c_{\text {ATP}}}{\partial t} +{\textbf {v}}\cdot \nabla c_{\text {ATP}} = D_{\text {ATP}} \nabla ^2 c_{\text {ATP}}\,, \end{aligned}$$

where $D_{\text {ATP}}$ is the diffusion coefficient of ATP in the plasma, and $c_{\text {ATP}}$ is the concentration of ATP in the plasma. Note that we only solve Eq. (7) for ATP concentration outside RBCs. The concentration of ATP in the cytoplasm of RBCs ($\sim$ 1 mM) is several folds larger than the ATP concentration in the plasma ($\sim$ 1 $\mu$M). Therefore, we consider the RBC as a large reservoir of ATP^25^. The detailed model of ATP release is given in following sections.

### ATP release model

According to Zhang et al.^24^, the release of ATP from RBCs follows two pathways (shown in Fig. 1): (1) the Px$_1$ hemichannels are activated when the local membrane shear stress exceeds a critical value and (2) the membrane deformation leads to activation of the CFTR, which up-regulates the Px$_1$ hemichannels. These two ATP pathways are mathematically expressed as

8 $$\begin{aligned} {p_\sigma = k_\sigma H\{\sigma _{mem}({\textbf {X}}(s)) - \sigma _c\}} \,, \end{aligned}$$

and

9 $$\begin{aligned} {p_c = \text {min}\left[ 1 + k_{\dot{c}}\{\dot{c}({\textbf {X}}(s))-\dot{c_c}\} H\{\dot{c}({\textbf {X}}(s))-\dot{c_c}\}, p_{\dot{c}\text {max}}\right] } \,, \end{aligned}$$

where $p_\sigma$ and $p_c$ are ATP release rates due to opening of Px$_1$ hemichannels and the CFTR amplification factor, respectively. $H(\cdot )$ is the Heaviside step function, $k_\sigma$ is a phenomenological coefficient, $\sigma _{mem}$ is the local membrane shear stress, and $\sigma _c$ is the critical shear stress required for opening of Px1 hemichannels. $k_{\dot{c}}$ is the slope of local membrane curvature change rate, $\dot{c}$ is the local curvature change rate (quantifying the degree of cell deformation), $\dot{c_c}$ is the critical curvature change rate for up-regulation of Px$_1$, and $p_{\dot{c}max}$ is the the maximum amount of CFTR. We define the flux on the outer surface of RBC^24^ as

10 $$\begin{aligned} \psi = p_\sigma p_c \,, \end{aligned}$$

which postulates that no ATP is released from the RBC when $\sigma _{mem} <\sigma _c$ and is not up-regulated by cell deformation (Eq. (9) ) when $\sigma _{mem} < \sigma _c$.

### Boundary conditions

The ATP release flux on the RBC membrane takes the form

11 $$\begin{aligned} D_{\text {ATP}} \frac{\partial c_{\text {ATP}}}{\partial {\textbf {n}}} = -\psi \,, \end{aligned}$$

where ${\textbf {n}}$ denotes the local normal vector pointing outward from the boundary to fluids, and the definition of $\psi$ is given in Eq. (10). Because of the strategic location of the endothelium (between the vascular lumen and tissues), it plays a critical role in maintaining the vascular tone. One of the important functions of EC is to maintain the concentration of ATP in the physiological range. This is achieved with help of ectonucleotidase enzymes. These enzymes act as a safeguard against unwanted activation of pathways or reactions with receptor due to the presence of abnormal ATP concentration^23^. Another function of EC is to sense the fluid flow. It is then transmitted to the cytoplasm for synthesis and liberation of signaling molecules such as ATP^39^. Several studies have been devoted^40–42^ to the understanding the role shear stress on the rise of cytoplasmic Ca$^{2+}$ concentration. One of the mechanisms may be the autocrine signaling^39,43,44^ i.e., EC releases ATP in response to the flow and these ATP molecules bind to the surface receptors of the same EC to initiate the Ca$^{2+}$ signaling reactions. These two functions of EC are mathematically expressed as the first and second terms in Eq. (12)^45^. The ATP flux on the surface of endothelium is written as

12 $$\begin{aligned} D_{\text {ATP}} \frac{\partial c_{\text {ATP}}}{\partial {\textbf {n}}} = \frac{V_{\text {max}} c_{\text {ATP}}}{K_m}- S_{\text {max}}\left[ 1- \left( \frac{-\tau _w}{\tau _0}\right) \right] ^3\,, \end{aligned}$$

where $V_{\text {max}}$ is the maximum enzymatic reaction velocity, $K_m$ is the Michaelis-Menten constant, $S_{\text {max}}$ is the maximum ATP release rate from the EC, $\tau _w$ and $\tau _0$ are the wall and reference shear stresses on the EC surface, respectively.

Once the velocity field is obtained by solving the Navier–Stokes equations (Eqs. (1), (2)), we use it to solve for ATP concentration field using the advection–diffusion equation (Eq. (7)). The advection–diffusion equation (Eq. (7)) is supplemented with boundary condition Eqs. (11), (12). Both fluid and ATP equations are solved numerically using an in-house lattice Boltzmann method (LBM) solver. The detailed parameter values for the ATP release model are given in Table 1, and these values are obtained from^2^ and^45^. The details of the numerical procedure can be found in^24,46^.

**Table 1:** ATP release parameters.

Parameters	Description	Value	References
$k_\sigma$	Phenomenological constant	1.75 $\times$ 10$^{2}$ nM $\mu$m s$^{-1}$	^25^
$\sigma _c$	Critical shear stress	0.05 Pa	^24^
$k_{\dot{c}}$	Slope of curvature change rate	6 $\times$ 10$^{-3} \mu$m s	^24^
$\dot{c_c}$	Critical curvature change rate	2 $\times$ 10$^{2}$ $\mu$m$^{-1}$s$^{-1}$	^24^
$p_{\dot{c}max}$	Maximum amount of CFTR	2.5	^24^
$D_{\text {ATP}}$	ATP diffusion constant	2.36 $\times$ 10$^{-10}$ m $^2/$s	^24^
$V_{\text {max}}$	Enzymatic reaction velocity	0.8 $\mu$mol m$^{-2}$ s$^{-1}$	^45^
$K_{\text {m}}$	Michaelis-menten constant	475 $\mu$M	^45^
$S_{\text {max}}$	Maximum ATP release rate	10$^{-3}$ nmol m$^{-2}$ s$^{-1}$	^45^
$\tau _0$	Reference shear stress	1 Pa	^45^

### Dimensionless numbers and some mathematical definitions

Dimensionless numbers are used to characterize the flow, the ATP distribution, and the ATP release level of RBCs.

1. The capillary number is defined as the ratio between the viscous stress of fluid and bending strength of the membrane
  13 $$\begin{aligned} Ca = \frac{\mu _{\text {ex}} \dot{\gamma _w} R_0^3}{\kappa } \,. \end{aligned}$$
  where $\dot{\gamma _{w}} = 4 v_{\text {max}}/{W}$ refers to the wall shear rate of the Poiseuille flow.
2. The confinement number is used to define the degree of confinement of a RBC by the gap of the channel. It is defined as
  14 $$\begin{aligned} Cn = \frac{2 R_0}{W} \,. \end{aligned}$$
3. To understand the role of ECs on the lumen ATP concentration, we define the spatial-averaged ATP concentration for a given time
  15 $$\begin{aligned} \overline{c}_{\text {ATP}} = \frac{1}{L W}\int _{0}^L \int _{-\frac{W}{2}}^{\frac{W}{2}} c_{\text {ATP}}(x,y,t) dy dx\,. \end{aligned}$$
  This will allow us to follow the global ATP concentration as a function of time in order to examine the evolution towards a steady state for ATP. The time-averaged ATP concentration along the channel is defined as
  16 $$\begin{aligned} <c_{\text {ATP}}>_{\text {transverse}} = \frac{1}{T}\int _{0}^T \frac{1}{L}\int _{0}^L c_{\text {ATP}}(x,y,t) dx dt \,. \end{aligned}$$
  This quantity will allow us to analyze the ATP spatial distribution in the *y*-direction, orthogonal to the flow direction. The time-averaged ATP concentration at the wall is defined as
  17 $$\begin{aligned} <c_{\text {ATP}}>_{\text {wall}} = \frac{1}{T}\int _{0}^T c_{\text {ATP}}(x,t) |_{\text {wall}} dt \,. \end{aligned}$$
  This is an important quantity since it is defined at the wall where ATP reacts with EC.
4. In a 2-D channel, the hematocrit is defined as the area occupied by RBCs (i.e., N$_c$ is the number of RBCs) to the area of the channel
  18 $$\begin{aligned} Hct = \frac{N_c A}{L W} \,. \end{aligned}$$

### Ca$^{2+}$ dynamics model of endothelial cell signaling

**Figure 2:**
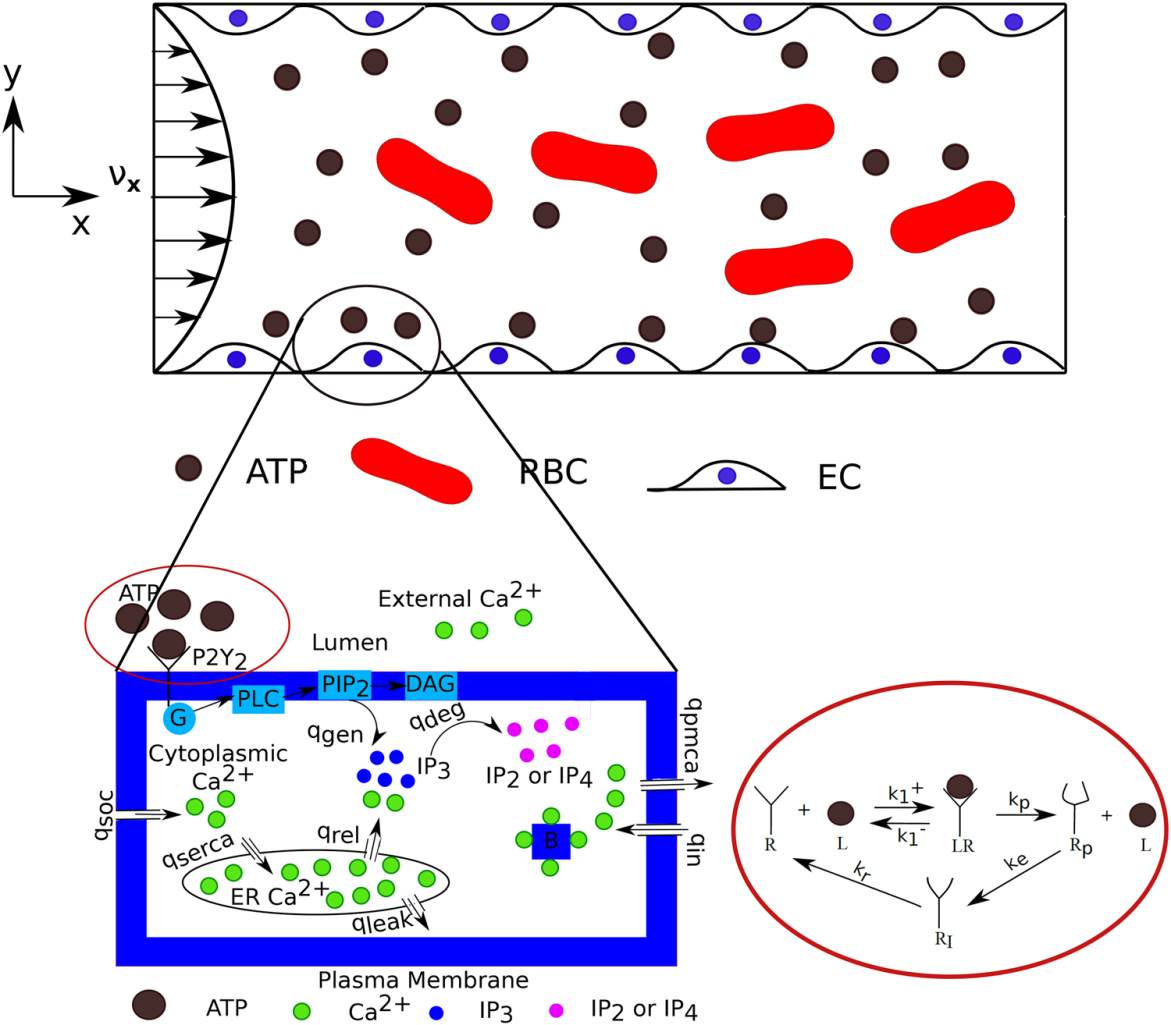
Schematic shows that red blood cells (RBCs) and endothelial cells (ECs) release adenosine triphosphate (ATP) molecules due to the application of parabolic-Poiseuille flow along x-direction. ATP is then transported to the wall-lined with ECs where ATP reacts with the surface receptors, in order to provoke the Ca$^{2+}$ signaling. Here, both walls of the vascular lumen are lined with ECs. The activation process of Ca$^{2+}$ signaling in an EC is shown in the enlarged Figure. This enlarged Figure depicts the activation of cascade of reactions and influx/efflux of Ca$^{2+}$ fluxes, across the plasma membrane (PM) and the endoplasmic reticulum (ER) membrane, due to the binding of ATP molecules with the purinergic receptors (P2Y$_2$). Words starting with *q* represent fluxes and expression for these fluxes are reported in Ca$^{2+}$ signaling subsection. The dynamics of P2Y$_2$ receptors is shown in the red colored enclosure. There are four states of a receptor: (**i**) unphosphorylated receptors, R, (**ii**) ligand-receptor complexes, LR , (**iii**) phosphorylated receptors, R$_p$, and (**iv**) internalized receptors, R$_I$. ATP concentration is denoted by L. $k_1^{+}$, $k_1^{-}$, $k_p$, $k_e$, and $k_r$ are the chemical reaction constants. Inositol trisphosphate (IP$_3$), inositol disphosphate (IP$_2$), inositol tetrakisphosphate (IP$_4$), G-protein (G), phospholipase C (PLC), phosphatidylinositol 4,5-bisphosphate (PIP$_2$), and diacylglycerol (DAG).

Recently, we have developed a model for calcium that ensures return to homeostasis in the presence of agonists^28^. The schematics of the model are recalled in Fig. 2. The basic new element (in comparison to previous literature where homeostasis was not achieved) in that model is the introduction of ATP-receptors desensitization.

The homeostatic model is described by 5 important variables^28^, namely unphosphorylated receptor ($[R_s]$), phosphorylated receptor ($[R_p]$), IP$_3$ ([$IP_3$]), cytoplasmic Ca$^{2+}$ ([$Ca_c$]), and store Ca$^{2+}$ ([Ca$_s$]). The evolution of these variables are obtained based on balance of fluxes across the membrane (PM and ER) as well as based on the chemical kinetics, and read

19 $$\begin{aligned} \frac{d[R_s]}{dt} = k_r [R_t] - \left( k_r + k_p \frac{[L]}{K_r + [L]} \right) [R_s] - k_r [R_p],\end{aligned}$$

20 $$\begin{aligned} \frac{d[R_p]}{dt} = k_p \frac{[L][R_s]}{K_r + [L]} - k_e[R_p],\end{aligned}$$

21 $$\begin{aligned} \frac{d\left[ IP_3\right] }{dt} = q_{\text {gen}}- q_{\text {deg}} , \end{aligned}$$

22 $$\begin{aligned} \frac{d\left[ Ca_c\right] }{dt} = \beta \left( q_{\text {rel}} - q_{\text {serca}} + q_{\text {leak}} + q_{\text {soc}} + q_{\text {in}}- q_{\text {pmca}} \right) , \end{aligned}$$

23 $$\begin{aligned} \frac{d\left[ Ca_s\right] }{dt} = v_r \left( q_{\text {serca}} - q_{\text {rel}} - q_{\text {leak}} \right) , \end{aligned}$$

where [*L*] (or is equivalent to the $c_{\text {ATP}}$ at the boundary) is the ATP concentration on the endothelium surface and $[R_t]$ is the total receptor concentration. Furthermore, the fluxes given in Eqs. (21)–(23) are defined below. The generation of IP$_3$ flux due to hydrolysis of the PIP$_2$ molecules by the PLC-enzymes is denoted by $q_{\text {gen}}$, and the degradation flux of IP$_3$ to inositol bisphosphate (IP$_2$) and inositol tetrakisphosphate (IP$_4$) due to the action of enzymes such as phosphates and kinases is denoted by $q_{\text {deg}}$. Other fluxes such as $q_{\text {rel}}$, $q_{\text {serca}}$, $q_{\text {leak}}$, $q_{\text {soc}}$, $q_{\text {in}}$, and $q_{\text {pmca}}$ are release of Ca$^{2+}$ from ER, uptake by the ER from cytoplasm, passive leak from ER, influx of the extracellular Ca$^{2+}$ to cytoplasm via the store-operated channel (SOC), passive leak of Ca$^{2+}$ from the extracellular space through PM, and pumping of cytoplasmic Ca$^{2+}$ to the extracellular space using PMCA, respectively. The value and meaning of each constant in Eqs. (19)–(20) as well as in the fluxes of Eqs. (21)–(23) are extracted from^28^.

24 $$\begin{aligned} q_{\text {gen}} =\gamma \frac{[Ca_c]}{K_1 + [Ca_c]}\,, \end{aligned}$$

where $\gamma = {\alpha } [G][(PIP_2)_t] / ( N_A V_{\text {EC}})$, $N_A V_{\text {EC}}$ is the product of the Avogadro’s number and the volume of an EC.

25 $$\begin{aligned}{}[G] = [G_t]\frac{\rho _r + \delta }{\rho _r + \delta + K_g} \equiv [G_t] \lambda \,, \end{aligned}$$

where $$K_g = k_d/k_a,$$

26 $$\begin{aligned} q_{\text {deg}} = k_2 \left[ IP_3\right] , \end{aligned}$$

27 $$\begin{aligned} q_{\text {soc}} = k_{\text {soc}} \frac{1}{\left( 1+\frac{\left[ Ca_s\right] }{K_{\text {soc}}}\right) ^n}\left[ Ca_{\text {ex}}\right] \left( \left[ Ca_{s}\right] (0)- \left[ Ca_s\right] \right) , \end{aligned}$$

28 $$\begin{aligned} q_{\text {pmca}} =k_8 \frac{\left[ Ca_c\right] ^2}{K_4^2 + \left[ Ca_c \right] ^2} , \end{aligned}$$

29 $$\begin{aligned} q_{\text {leak}} = k_5 [Ca_s] , \end{aligned}$$

30 $$\begin{aligned} q_{\text {serca}} = k_4 \frac{\left[ Ca_c\right] ^2}{K_{3}^2+ \left[ Ca_c \right] ^2},\end{aligned}$$

31 $$\begin{aligned} q_{\text {rel}} = k_3 \frac{\left[ IP_3\right] ^3}{K_2^{3} + \left[ IP_3\right] ^3} {\frac{ K_{i,Ca_c}^3}{\left[ Ca_c\right] ^{3} + K_{i,Ca_c}^{3}} } Ca_s , \end{aligned}$$

32 $$\begin{aligned} q_{\text {in}} = k_8 \frac{([Ca_c](0))^2}{K_4^2 + ([Ca_c](0))^2}, \end{aligned}$$

33 $$\begin{aligned} \beta = \left\{ 1 + \frac{\left[ B_t\right] \frac{k_7}{k_6}}{\left( \frac{k_7}{k_6} + \left[ Ca_c\right] \right) ^2}\right\} ^{-1}, \end{aligned}$$

where $\beta$ is a buffer factor. The buffer factor is obtained using the rapid equilibrium approximation i.e., the binding and unbinding reactions of free Ca$^{2+}$ and buffer proteins are governed by the fast-kinetics.

## Results and discussion

We have explored the effect of three main parameters on ATP and calcium signaling, namely hematocrit *Hct* (2–45%), capillary number *Ca* (9–90), and degree of confinement *Cn* (0.2–0.8). These ranges are consistent with those known for microcirculation^25^, where most of transport processes such as oxygen, nutrients, ATP, and metabolic wastes are carried out. We recall that both diffusion and advection of ATP are taken into account. ATP is released by RBCs, albeit ECs also contribute to some extent (see below) to its release. RBCs are transported by the flow and both their shapes and their spatio-temporal organization are not known a priori, and are obtained by solving fluid equations subject to boundary conditions (see Eqs. (1), (2)). Once ATP is released and reacts with EC receptors, Ca$^{2+}$ generation from ECs is triggered.

**Figure 3:**
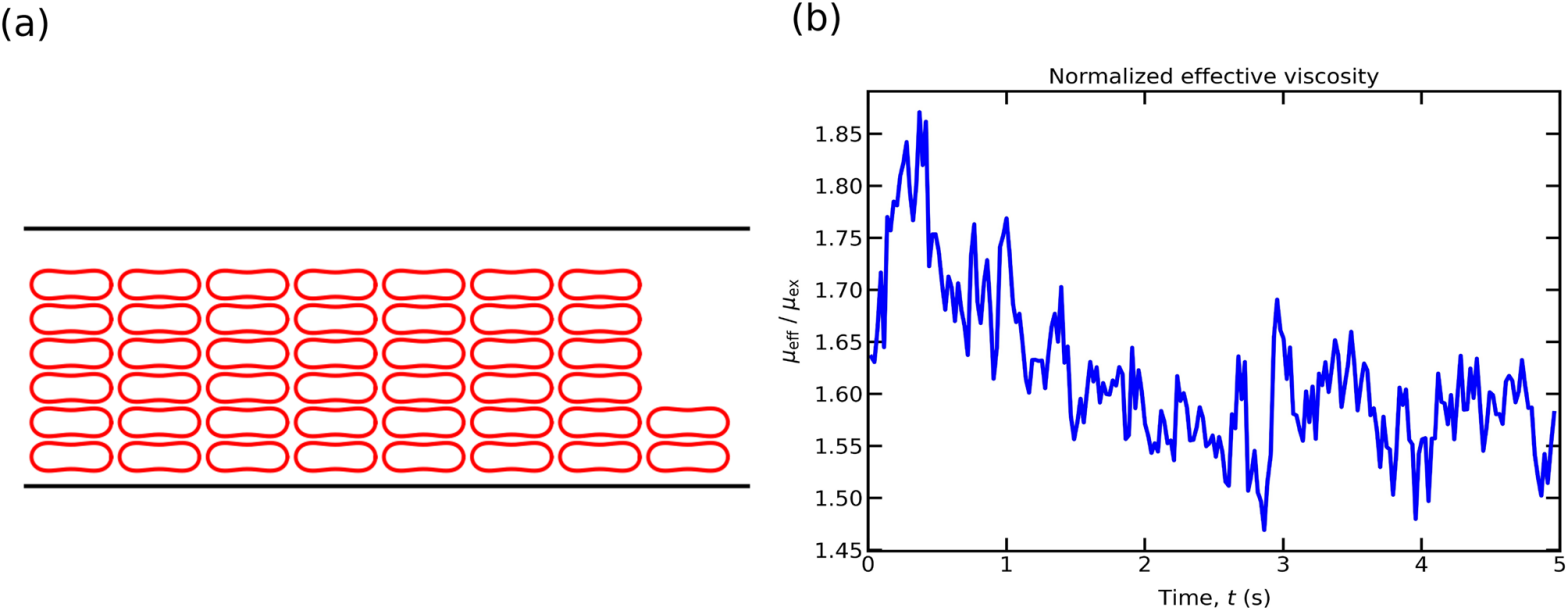
(**a**) Initial spatial arrangement of RBCs ($Hct = 45.9 \%$) for $Cn = 0.2$; and (**b**) the evolution of the ratio of the effective viscosity of the suspension to the viscosity of the plasma with time corresponding to $Hct = 45.9 \%$, $Cn = 0.2$, and $Ca = 90$.

Firstly, we determine the distribution of ATP throughout the channel as well as on the endothelium surface (i.e., bottom and top walls), by solving the hydrodynamic equations for RBCs (Eqs. (1), (2)) coupled with advection–diffusion equations for ATP (Eq. 7). The physical Reynolds number (i.e., $Re = \rho \dot{\gamma _w} R_0^2/\mu _{\text {ex}}$) is at most of the order of 10$^{-4}$. In our numerical study, we set the simulation Reynolds number to 0.1, for all the simulations. This helps accelerate the simulation speed without compromising the accuracy of simulation results^47^. As an initial state we select, arbitrarily, a regular arrangement of RBCs in the channel (see Fig. 3(a)). Note that another initial configuration, such as random positions of RBCs, leads to the same results. In our simulation the blood flow stream reaches steady state configuration in about 2.5 s (for the parameters chosen in the caption of Fig. 3a). In order to show the evolution of RBCs dynamics with time, we present in Fig. 3b the behavior of the effective viscosity, which is defined by

$$\eta _{\text {eff}} = \frac{\mu _{\text {ex}} Q}{Q^{(\text {ssp})}},$$

where $Q^{(\text {ssp})}$ is the average vesicle suspension flux and *Q* is the flux of plasma (without vesicles) at the same pressure gradient as $Q^{(\text {ssp})}$. It is seen in that figure (Fig. 3b) that the viscosity reaches its permanent regime in about 2.5 s. At the same time as RBCs evolve in the flow, we also determine the ATP distribution in the channel. Note that we expect a difference in the timescale required to achieve a steady-state for a suspension of capsules (a quite well adopted model for RBCs), due to membrane viscosity, which is large as compared to the internal viscosity and that of the suspending fluid^48,49,51^. Prado et al.^50^ estimated the two-dimensional membrane viscosity from experiments and reported a value $\mu ^{\text {2D}}_{\text {mem}} \simeq 0.6-0.85 \times 10^{-7}$ N s/m. In a simulation study by Gürbüz et al.^48^ a value of membrane viscosity $\mu ^{\text {2D}}_{\text {mem}} \sim 10^{-6}$ N s/m was considered, which is about ten times larger than that measured experimentally^50^. The simulation study by Guglietta et al.^49,51^ considered a wide range of membrane viscosity. For a value of membrane viscosity $\mu ^{\text {2D}}_{\text {mem}} = 0.6 \times 10^{-7}$ N s/m (consistent with that reported in experiments^50^, for which the Boussinesq number, *Bq* , = $\mu ^{\text {2D}}_{\text {mem}} / (R_0 \mu _{\text {ex}}) = 10$), it is found that the time required for single capsule or RBC to reach a steady state configuration in the presence of shear flow with that value of membrane viscosity is approximately 1.7-1.9 times longer than than that obtained without membrane viscosity, $Bq = 0$ or $\mu ^{\text {2D}}_{\text {mem}} = 0$ N s/m (see Fig. 10(a) in Guglietta et al.^51^ and Fig. 2 in Guglietta et al.^49^). Given the fact that our simulation of suspension requires about 2.5 s to reach a steady-state, we expect the corresponding time in the presence of membrane viscosity to be of about 4 s, significantly smaller than the time required for the development of calcium peak (which lies in the 10-30 s range). Therefore the separation of time scales (that corresponding to RBCs reaching steady-state, and that corresponding to calcium kinetics) seems to be reasonably legitimate. For this reason, we first solved for blood flow in order to determine the ATP distribution on the walls after RBCs have reached their steady-state configuration. We then inject this information into the calcium model. We repeated the same procedure for each set of parameters such as capillary number, confinement, and hematocrit.

Note that the magnitude of Ca$^{2+}$ concentrations in the blood lumen is several orders greater than that of the cytoplasmic Ca$^{2+}$ concentration^52^ . As a consequence, the lumen Ca$^{2+}$ concentration is taken as a homogeneous and infinitely large reservoir of Ca$^{2+}$ (term in Eq. (28)).

### Enzymatic activity of nucleotidases

**Figure 4:**
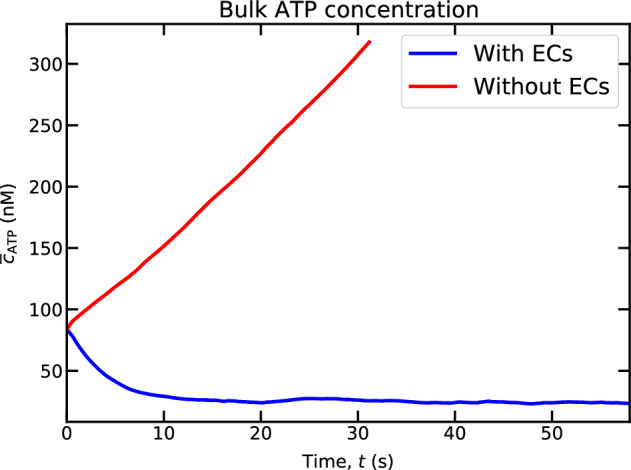
The evolution of bulk ATP concentration with time in the presence and absence of endothelium ($Ca = 22.5$, $Cn = 0.4$, and $Hct = 16.7 \%$).

In addition to transmitting chemical or mechanical cues from the blood lumen into the cytoplasm of cell, ECs play a vital role in maintaining blood ATP concentration within the physiological range. As discussed in Subsection Boundary conditions, ectonucleotidase enzymes act as a safeguard against unwanted or abnormal activation of pathways or reactions with receptors due to uncontrolled ATP concentration in the vascular lumen^23^. These enzymes hydrolyze ATP into ADP and eventually, ADP into AMP and AMP into AD. As a result, the potential harmful effects of ATP are avoided. A recent study^25^ did not taken into account the role of ECs, so that ATP released by RBCs increased with time without bound. We include here the role of ECs in our model as a prescribed boundary condition (i.e., Eq. (12)). To understand the role of ECs in lumen ATP concentration, we have analyzed the behavior of the spatially-averaged ATP concentration with time. Interestingly, we found that ATP concentration reaches a steady-state in the lumen (Fig. 4). This result is exactly due to the balance of ATP release fluxes from RBCs and ECs with hydrolysis of ATP by the ectonucleotidase enzymes. We also demonstrate the behavior of ATP in the absence of endothelium intervention in that figure. Here, we have started the simulation with an initial ATP concentration higher than the steady ATP concentration.

### Wall ATP concentrations

**Figure 5:**
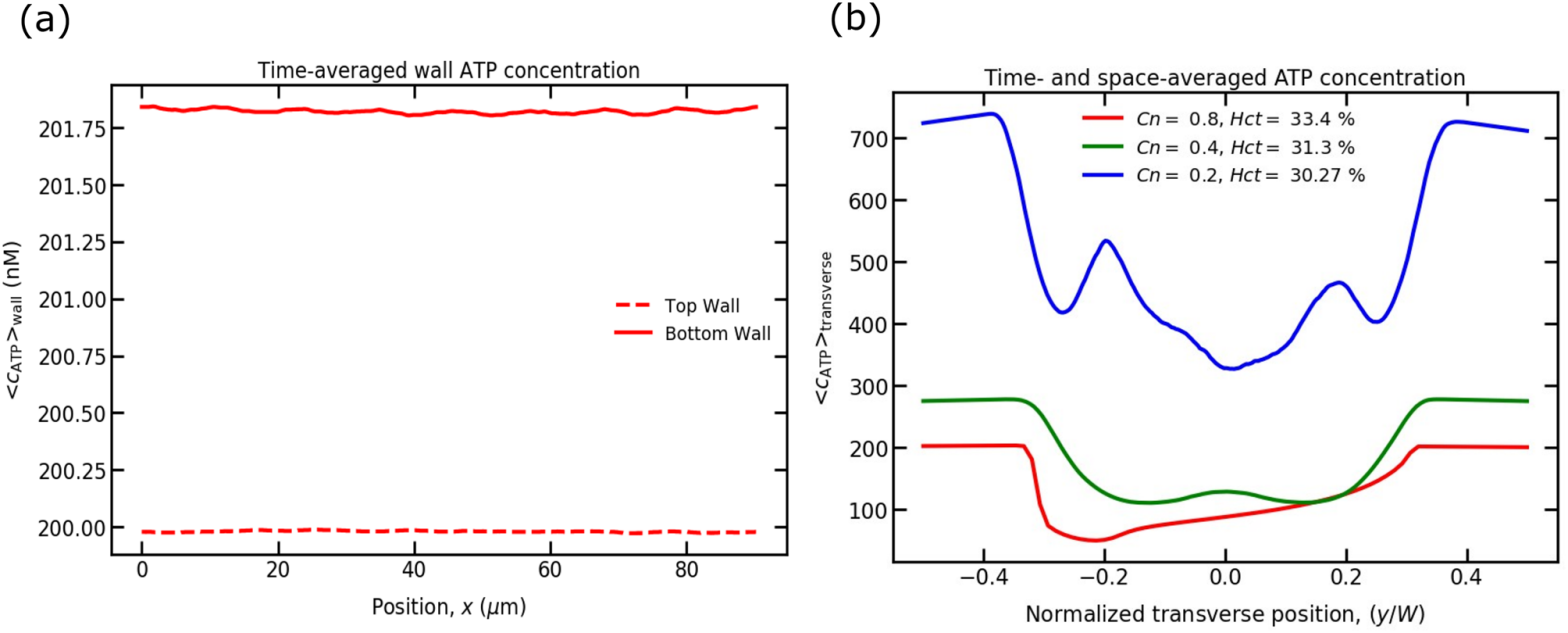
ATP concentrations are reported for $Ca =$ 90. (**a**) Time-averaged wall ATP concentrations for $Cn =$ 0.8 and $Hct =$ 33.4 $\%$; and (**b**) the time- and space-averaged ATP concentration across the width of the channel for various *Cn* and *Hct*.

Prior to coupling the response from the endothelium, i.e., studying Ca$^{2+}$ signaling, we first analyze how ATP concentrations vary along the top and bottom endothelium walls for different degrees of confinement, *Cn*, in a straight channel. There are almost no variation in ATP concentrations along both walls of the endothelium (Fig. 5a), as expected. The ATP concentration difference between the two walls is quite small. This is due to fact that after a long time has elapsed in the simulation, the RBCs in the channel occupy equal distances from the walls. As a consequence, the RBCs near to the two walls experience equal shear stress and ATP release. This leads practically to the same ATP concentration for both walls, as shown in Fig. 5a. For this reason, in next sections we will only consider the maximum ATP concentration from the bottom wall, which will be used as an input for Ca$^{2+}$ signaling activation in ECs, for various *Cn*, *Ca*, and *Hct*.

To understand how the ATP concentration varies across the channel for different *Cn* with a given *Hct* and *Ca*, we represent the transverse time- and space-averaged ATP concentration, as shown in Fig. 5b. We find that the ATP concentration is higher near the walls than that in the core region. This is owing to the facts: (1) ATP concentration is 0 in the area occupied by RBCs. Therefore, the overall ATP concentration in the core region is lower than that near the walls. (2) RBCs spread closer to the walls due to cell-cell hydrodynamic interactions. The high shear stress near the walls induces and increase of ATP release, resulting in a higher ATP concentration. This high concentration of ATP close to the walls is beneficial for calcium signaling in ECs, which we will discuss in upcoming sections. With varying *Cn*, we observe that the ATP concentration is higher in wider channels than in smaller width channels, as shown in Fig. 5b. This can be explained by the following argument: the number of RBCs is inversely proportional to *Cn* for a constant *Hct*. Thus, there are more RBCs in the case where $Cn = 0.2$ than in in the cases $Cn = 0.4$ and 0.8, leading to a greater accumulated ATP release by RBCs and consequently a higher ATP concentration. The ATP concentrations both near the walls and core regions are higher for the case $Cn = 0.2$ compared to the case $Cn = 0.4$ and 0.8. Similarly, the ATP concentrations both near the walls and core regions are higher in $Cn = 0.4$ than in $Cn = 0.8$; however, the differences are minimal.

### Effect of capillary number

**Figure 6:**
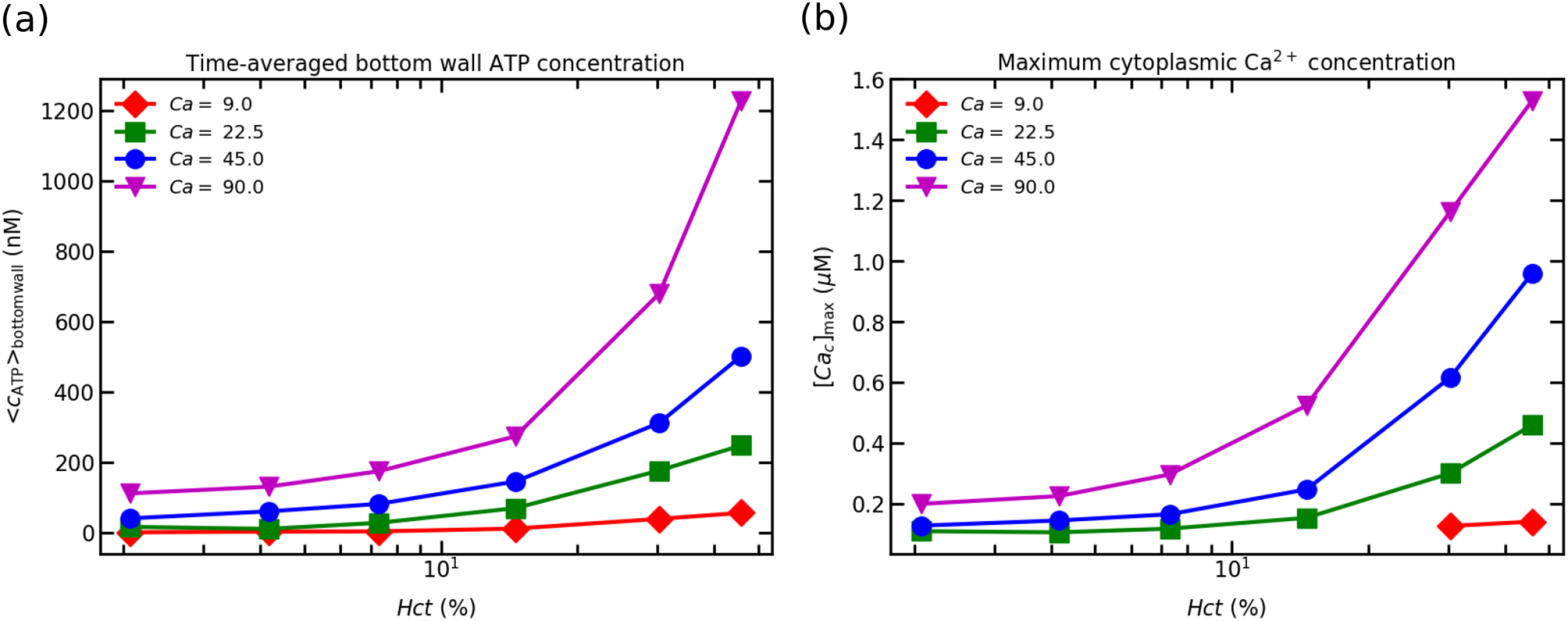
These results are obtained for $Cn =$ 0.2 on the bottom wall of a 2-D channel: (**a**) maximum ATP concentrations on the bottom wall with varying *Hct* concentrations for various *Ca*; and (**b**) maximum cytoplasmic Ca$^{2+}$ concentrations (transient peaks) obtained from the maximum ATP concentrations for different *Ca*.

We have carried out several simulations by choosing different *Ca* while fixing confinement (*Cn*) to 0.2. In real microvascular networks, RBCs experience different wall shear rates when traversing through the vessels of the same diameter. To emulate such a fact in the microvasculature, we have performed simulations by varying *Ca*. In numerical simulations, we are, in principle, in the Stokes limit (zero *Re*), but the lattice Boltzmann method (LBM) introduces inherently a finite Reynolds number, that must be kept small enough. The real *Re* is about $10^{-4}$, and a choice of this value in the LBM would make computation quite prohibitive. There is a consensus in the literature that fixing $Re=0.1$ helps accelerate the simulation speed without compromising the accuracy of simulation results^47^ . Since we aim to keep the same *Re* for all simulations, we have fixed the flow speed (to have $Re=0.1$) to the same value for all simulations, while varying the bending rigidity to explore different values of *Ca*. Fig. 6a shows the maximum time-averaged bottom wall ATP concentration. The concentration of ATP at the wall surface increases with an increase in *Hct* concentration for each *Ca*. From Fig. 6a, the following conclusions can be drawn: (1) the maximum ATP release level (ATP releasing capacity of a RBC to its maximum ATP releasing capacity), as reported by^25^, does not play a vital role in deciding the quasi-steady ATP concentration, (2) the final ATP concentration depends on *Hct* i.e., high *Hct* means the higher cumulative ATP concentration and total ATP release level (3) $Ca = 9$ is unable to cause any significant change in the concentration of ATP for different *Hct*. This is due to fact that the critical shear stress required to activate Px$_1$ hemichannels on the RBC membrane is not attained. Consequently, the concentration of ATP does not change significantly, (4) furthermore upon increasing of *Ca* (i.e., $Ca = 22.5$ and 45), more Px$_1$ hemichannels are opened due to the high shear stress experienced by RBC membrane. This membrane shear stress is higher than the critical shear stress required to activate these hemichannels. Further, the ATP released from ECs does not play a significant role to lumen ATP concentration for $Ca = 22.5$ and 45. And also, the contribution of CFTR on the up-regulation of Px$_1$ hemichannels is also negligible due to the small deformation of the RBC membrane, (5) with further increase in the flow strength i.e., *Ca* ($Ca = 90$), almost all Px$_1$ hemichannels channels are activated as reported by Gou et al.^25^. In the meantime, these channels are also up-regulated by CFTRs, which are activated due to the deformation of the membrane at $Ca =90$. Furthermore, the shear stress experienced by the EC is sufficient to contribute to the lumen ATP concentration. Another important remark is that for high *Hct* concentration and $Ca = 90$ more RBCs are pushed towards the wall due to cell–cell interaction, resulting in higher shear stress experienced by RBCs and higher ATP release, and (6) the ATP concentration at the wall is linear for the dilute concentrations (i.e., up to 10 $\%$). After that regime, the wall ATP concentration becomes nonlinear with increasing in *Hct*.

Finally, we provide ATP wall concentration as an input to solve Ca$^{2+}$ signaling Eqs. (19 –23). We present the maximum cytoplasmic Ca$^{2+}$ concentration (Ca$^{2+}$ peak) in Fig. 6b. The response of an EC, the cytoplasmic Ca$^{2+}$ concentration, follows the same trend as the wall ATP concentration (see Fig. 6a). The cytoplasmic Ca$^{2+}$ concentration rises above the homeostatic concentration, 0.1 $\mu$M for all *Ca* and *Hct*. However, the response for $Ca =$ 9 is very negligible in comparison to other *Ca*. This is due to the fact that the low concentration of ATP on the surface of the endothelium wall is unable to produce IP$_3$ molecules in sufficient quantities by phosphorylating free receptors. As a consequence, the concentration of IP$_3$ molecules required to activate IP$_3$R (inositol trisphosphate receptor) channels present on the membrane of ER is not sufficient to mobilize Ca$^{2+}$.

### Effect of confinement

**Figure 7:**
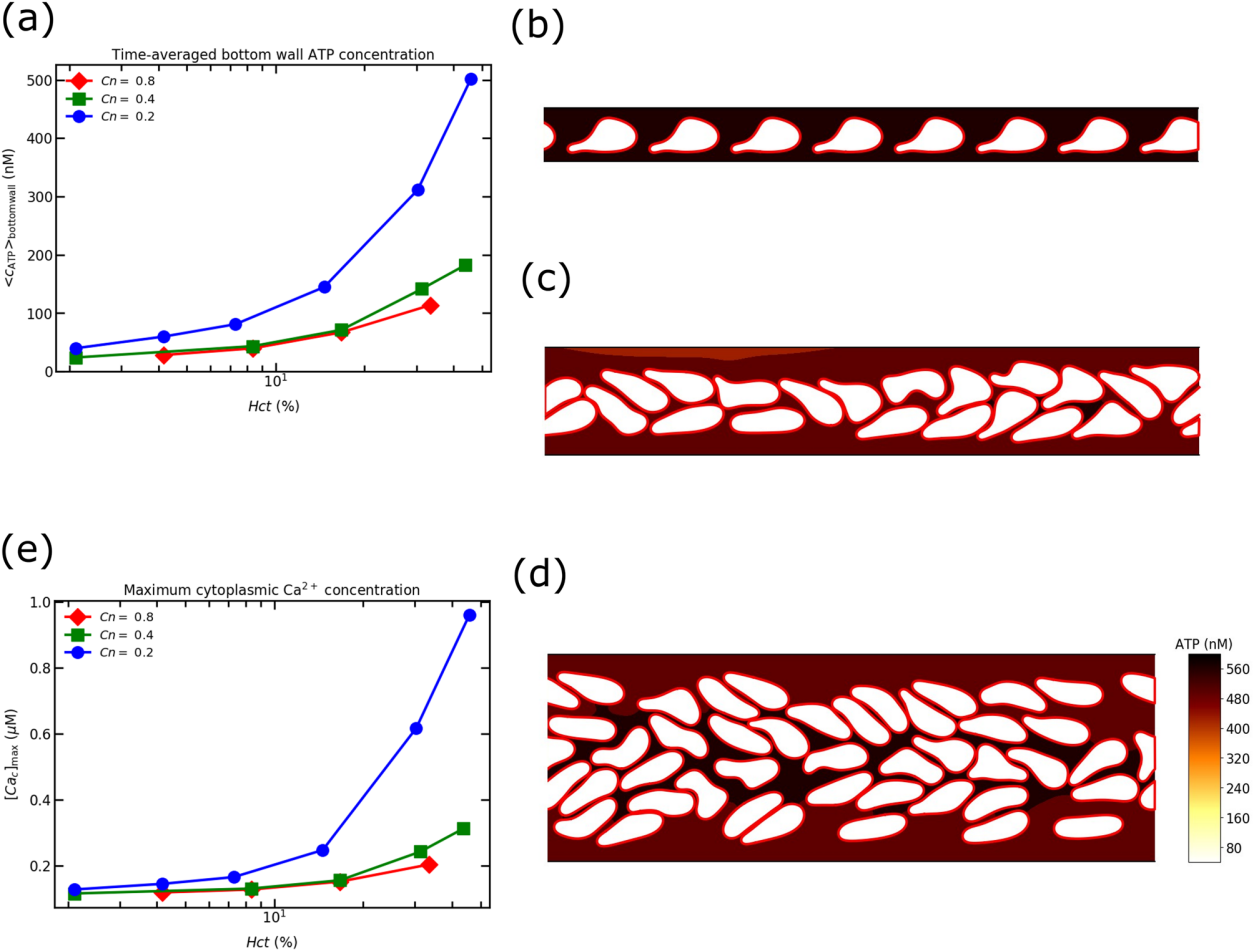
(**a**) Maximum ATP concentration on the bottom wall by varying *Hct* concentration for various *Cn*; (**b**) spatial concentration of ATP at $Cn =0.8$, $Ca = 45$, $Hct = 33.4 \%$, and $t = 18.56$ s; (**c**) spatial concentration of ATP at $Cn =0.4$, $Ca = 45$, $Hct = 43.4 \%$, and $t = 26.9$ s; (**d**) spatial concentration of ATP at $Cn =0.2$, $Ca = 45$, $Hct = 45.9 \%$, and *t* = 25.87 s; and (**e**) the maximum cytoplasmic Ca$^{2+}$ concentration (transient peak) obtained from the corresponding maximum ATP concentration for various *Cn*. All simulations are carried out for $Ca =45$. The Color bars are removed for $Cn = 0.8$ and 0.4 due to the absence of visible ATP concentration gradients in plasma.

In this section, we study the effect of *Cn* on ATP and the cytoplasmic Ca$^{2+}$ concentration for $Ca =$ 45. The results obtained with increasing *Cn* are similar to the observations made in previous subsection. In addition, the following observations can be drawn from Fig. 7a: (1) interestingly, the ATP concentrations for $Cn =$ 0.4 and $Cn=$ 0.8 are very close to each other. There are no significant differences in the results within the dilute regime (upto $Hct = 10 \%$). However, a difference is visible beyond the dilute regime. Gou et al.^25^ have reported that the ATP release level per RBC for $Cn = 0.8$ is always higher than the ATP release level for $Cn = 0.4$ for all *Hct* concentrations. This is due to the fact that at $Cn =0.8$, RBCs flow close to the wall (see Fig. 7b) like a train for different *Hct* concentrations. As result, the RBCs experience higher membrane shear stress, which helps increase the ATP release level. It has already been proven that the shear stress-dependent ATP release through Px$_1$ hemichannels predominates over the CFTR amplification factor. The CFTR amplification factor is associated with RBC deformation. This conclusion was drawn based on measurements of the averaged shear stress per RBC and the averaged rate of curvature change per RBC for different flow strengths. Specifically, it was observed that, particularly at high enough *Ca*, the average shear stress exceeds the critical shear stress required to activate Px$_1$ hemichannels. However, the average rate of local curvature change remains below the critical rate of curvature change in the majority of configurations^24,25^ (except when a cell hits a bifurcation, a geometry which is outside the present scope). Consequently, shear stress-dependent ATP release assumes greater significance than RBC deformation. However, for the same length of channel the number of RBCs in $Cn = 0.4$ is greater than in $Cn = 0.8$, resulting in a higher total ATP release level in $Cn = 0.4$ than the total ATP release level in $Cn = 0.8$; and (2) the rapid increase in ATP concentration is observed for $Cn =$ 0.2. The reason is due to the development of the complex cell-cell interactions and dispersion of RBCs in the channel. As a result, RBCs experience higher shear stress and membrane deformation which results in greater ATP concentration in contrast to other values of $Cn = 0.8$ and 0.4. Fig. 7c shows that at $Cn = 0.4$, the RBCs are oriented in an order and close to the center of the channel. In contrast, at $Cn = 0.2$ the RBCs are randomly oriented and distributed in the channel, as shown in Fig. 7d.

There is not a significant visible ATP gradient in the plasma for $Cn = 0.8$ and 0.4, as shown in Fig. 7b,c. However, a visible ATP gradient does exist for $Cn = 0.2$, as shown in Fig. 7d. The concentration of ATP between the spaces between RBCs is higher than in the cell-free layer (CFL) regions for $Cn = 0.2$. Due to cell-cell interactions, the released ATP from the core region does not diffuse freely into the CFL regions, where RBCs serve as obstacles.

Consequently, the concentration of ATP in the free spaces between cells remains higher than in the CFL regions. At higher RBC concentrations, for $Cn = 0.4$ and 0.8, RBCs flow in double and single-file trains, respectively (shown in Fig. 7b,c). In both cases, the core regions are crowded, with minimal free spaces between cells, and the CFL layers are uniform.

The ATP release is higher near the walls in these confinements, as cells are very close to the walls where shear stress is highest. The released ATP near the walls accumulates in the CFL regions and is balanced by the hydrolysis of enzymes present on the endothelial wall surface (shown in Fig. 5b), resulting in a stable ATP concentration. Therefore, due to the small CFL thickness and velocity in the wall vicinity, the ATP in the CFL regions can be assumed to be diffusion dominated, at least for $Cn = 0.4$ and 0.8.

The maximum cytoplasmic Ca$^{2+}$ concentration observed in an EC follows the same trend as ATP concentration (Fig. 7e). The maximum cytoplasmic Ca$^{2+}$ increases monotonously with increase in *Cn* and *Hct*. Moreover, an increase in the maximum cytoplasmic Ca$^{2+}$ concentration may lead to propagation of Ca$^{2+}$ signals from region of high cytoplasmic Ca$^{2+}$ concentration to lower cytoplasmic Ca$^{2+}$ concentrations. A real vascular network has different vessel sizes as well as different local hematocrits. According to the results of Fig. 7e, it may happen that ATP concentration reaches the threshold value for calcium signaling in some locations (e.g. with high enough *Hct*, or large shear stresses). This naturally raises the question of calcium propagation in vascular network. It is hoped to investigate this matter in a future work. Here, we will first address the question of the transient peak time of the cytoplasmic Ca$^{2+}$ concentration, which will help clarify the coordinating cellular function for calcium propagation in a vascular network.

### Cytoplasmic Ca$^{2+}$ peak time

**Figure 8:**
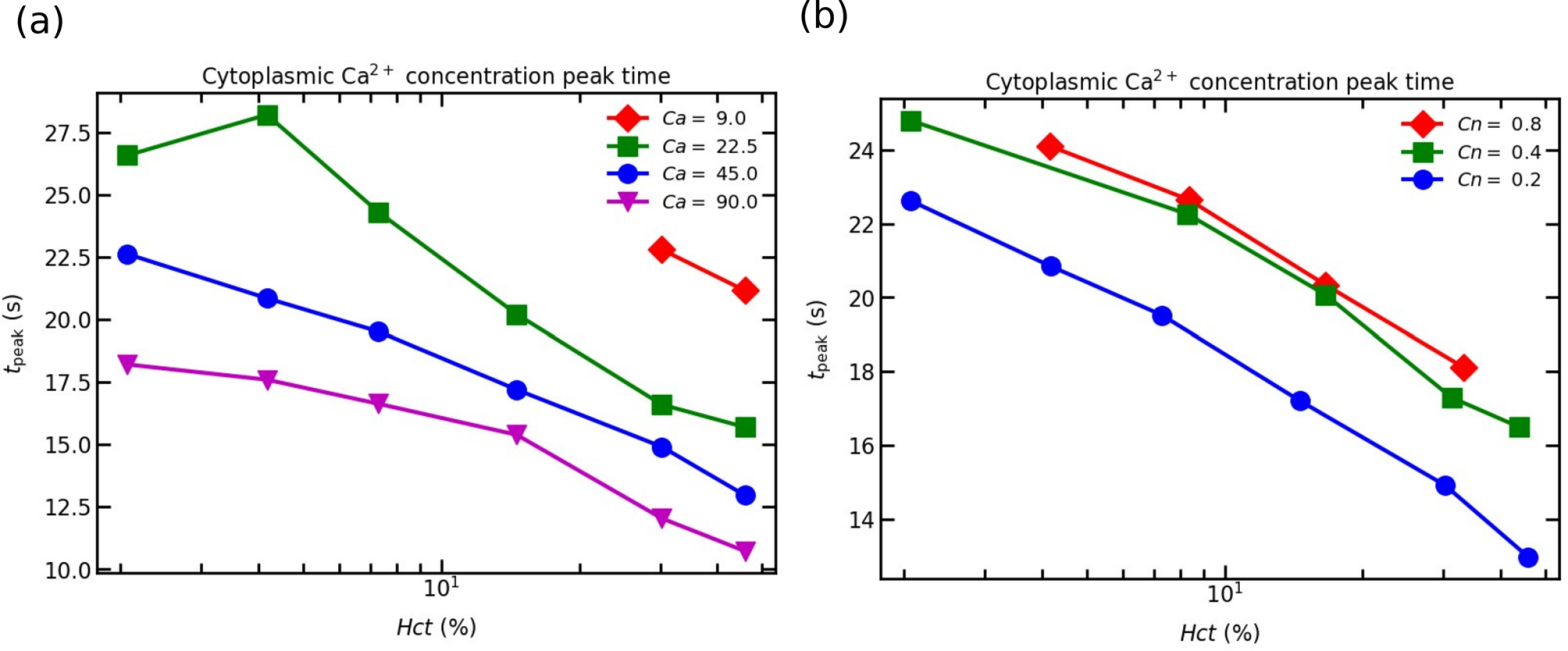
The cytoplasmic Ca$^{2+}$ peak in an EC: (**a**) the peak time obtained by varying *Hct* concentration for different *Ca* and $Cn =$ 0.2; and (**b**) the peak time obtained by varying *Hct* for different *Cn* and $Ca =$ 45.

In order to understand the possibility of intercellular communication in the vascular wall, we present the transient peak time of the cytoplasmic Ca$^{2+}$. The response of an EC, the maximum cytoplasmic Ca$^{2+}$ concentration, is dose-dependent i.e., high ATP concentration leads to high cytoplasmic Ca$^{2+}$ concentration. The dependence between ATP and cytoplasmic Ca$^{2+}$ is sigmoidal^28^ due to the fixed quantity of production of IP$_3$ molecules. Furthermore, the high ATP concentration leads to a decrease in the cytoplasmic Ca$^{2+}$ transient time. In contrast, the low ATP concentration leads to a slower response from an EC. This is elucidated in Fig. 8. Figure 8a shows the dependence of the calcium peak time on *Hct* and *Ca*. The cytoplasmic transient time for a given *Ca* decreases with an increase in *Hct* concentration (see Fig. 8(a)). Similarly, the transient peak time decreases monotonically with an increase in Ca. The peak time difference between two different values of *Ca* and a given *Hct* concentration varies between 2 and 5 s. As a result calcium can propagate from one site to another, both downstream and upstream. This helps coordinating cellular functions, such as cellular proliferation, wound healing, vasodilation, etc. Since calcium propagation speed (in the range of $10 \mu$m/s^53–55^) is lower than the blood flow speed (in the range of mm/s in microcirculation), the vasodilation is controlled upstream only.

Figure 8b shows that for a given *Hct* concentration at $Cn =$ 0.4 and 0.8, there is no significant difference in the cytoplasmic Ca$^{2+}$ peak time. The cytoplasmic Ca$^{2+}$ peak time at $Cn =$ 0.2 is much faster than the transient peak time at $Cn =$ 0.4 and 0.8. This is due to the fact that there exists higher ATP concentration at $Cn = 0.2$ (see Fig. 7a). Therefore, there is a possibility of signal transfer from large width channel (weakly confined channel) to small width channels (strongly confined channels). In experiments with the vascular network stimulating a region with an agonist concentration^56–58^, they observed propagation of Ca$^{2+}$ to the downstream smaller vessel diameters. Therefore, our findings would be useful for understanding Ca$^{2+}$ propagation both in-vitro and in-vivo vessel networks.

### Calcium wave propagation

Intercellular Ca$^{2+}$ waves are responsible for coordinated functions of the cell activities such as proliferation or division of cells and transmission of vasodilation signals to upstream and downstream in blood vessel^54^. To demonstrate the propagation of a Ca$^{2+}$ wave from an EC stimulated by a high ATP concentration to its neighboring ECs, we perform a simple study by considering a group of horizontally aligned ECs. These ECs communicate with each other via gap junctions, which allow Ca$^{2+}$ and IP$_3$ to transfer to their neighboring ECs^55^. This is one of several mechanisms through which Ca$^{2+}$ and IP$_3$ diffuse in vascular wall. However, there are other mechanisms through which Ca$^{2+}$ propagation occurs in vascular wall. The propagation of action potential through ECs and SMCs from the stimulated site (high ATP concentration region)^59^ to the neighboring ECs and SMCs. The release of ATP from an EC into the extracellular space due to the mechanical stimulation and then diffusion of ATP results in the propagation of Ca$^{2+}$ waves over several distances in vascular wall^60,61^.

To investigate intercellular Ca$^{2+}$ waves in the current model, we begin with the same model equations given in Ca$^{2+}$ signaling subsection, and we additionally include the spatial variation of IP$_3$ and the cytoplasmic Ca$^{2+}$ concentrations by incorporating the diffusion of IP$_3$ and cytoplasmic Ca$^{2+}$ in Eqs. (21)–(22). So, the new modified equations (Eqs. 21, 22) become

34 $$\begin{aligned} \frac{\partial \left[ IP_3\right] }{\partial t} = D_{IP_3}\frac{\partial ^2 [IP_3]}{\partial x^2} + q_{\text {gen}}- q_{\text {deg}} , \end{aligned}$$

35 $$\begin{aligned} \frac{\partial \left[ Ca_c\right] }{\partial t} = D_{ca}\frac{\partial ^2 [Ca_c]}{\partial x^2} + \beta \left( q_{\text {rel}} - q_{\text {serca}} + q_{\text {leak}} + q_{\text {soc}} + q_{\text {in}}- q_{\text {pmca}} \right) , \end{aligned}$$

where $D_{IP_3}$ is the diffusion coefficient of IP$_3$ (i.e., 280 $\mu$m$^2$/s^55^), and $D_{ca}$ is the effective cytoplasmic diffusion coefficient of Ca$^{2+}$ (i.e., 20 $\mu$m$^2$/s^55^). The boundary conditions at the EC-EC interface are

36 $$\begin{aligned} D_{IP_3}\frac{\partial \left[ IP_3\right] }{\partial x} = P_{IP_3}\left( [IP_3]_{+} - [IP_3]_{-}\right) , \end{aligned}$$

37 $$\begin{aligned} D_{ca}\frac{\partial \left[ Ca_c\right] }{\partial x} = P_{ca}\left( [Ca_c]_{+} - [Ca_c]_{-}\right) , \end{aligned}$$

where $P_{IP_3}$ and $P_{ca}$ are the permeability rate constants for IP$_3$ and cytoplasmic Ca$^{2+}$, which are estimated to 0.05 $\mu$m/s and 5.0 $\mu$m/s, respectively^55^. $[IP_3]_{+}$ and $[IP_3]_{-}$ are IP$_3$ concentrations on either sides of the boundary, and $[Ca_c]_{+}$ and $[Ca_c]_{-}$ are cytoplasmic Ca$^{2+}$ concentrations on either sides of the boundary.

**Figure 9:**
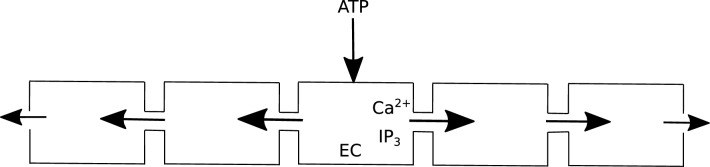
Schematic shows that a group of ECs are horizontally aligned and the middle EC is stimulated to a ATP concentration. Following stimulation, IP$_3$ and cytoplasmic Ca$^{2+}$ diffuse through gap junctions into the neighboring ECs. IP$_3$ molecules then release sequestered Ca$^{2+}$ from neighboring ECs. The propagation of Ca$^{2+}$ ions continues until IP$_3$ molecules are able to activate IP$_3$R (inositol trisphosphate receptor) present on ER membrane. The width of the arrow indicates the strength of propagation of IP$_3$ molecules and cytoplasmic Ca$^{2+}$ into the neighboring ECs.

The concentration variation in the lateral direction is smaller than that in the longitudinal direction, implying that molecular diffusion occurs more rapidly in the lateral direction. For the purpose of demonstration, we limit our investigation to a cluster of 9 endothelial cells (ECs). Specifically, there are 4 ECs on each side of the stimulated EC. For instance, we locally stimulate the middle EC (indicated by the downward arrow in Fig. 9) with a concentration of 1 $\mu$M ATP. This localized stimulation triggers signal propagation on both sides of the stimulated EC. The stimulated EC is designated as “EC0”, and the ECs to its right are labeled as “EC1”, “EC2”, “EC3”, and “EC4”. Similarly, the ECs to its left are named “EC5”, “EC6”, “EC7”, and “EC8”. We maintain the constants as provided in^28^, with the exception of altering the values of $K_r$ and $k_2$ to 0.5 $\mu$M and 0.02 s$^{-1}$ respectively, to assess their impact on the speed of Ca$^{2+}$ wave propagation.

**Figure 10:**
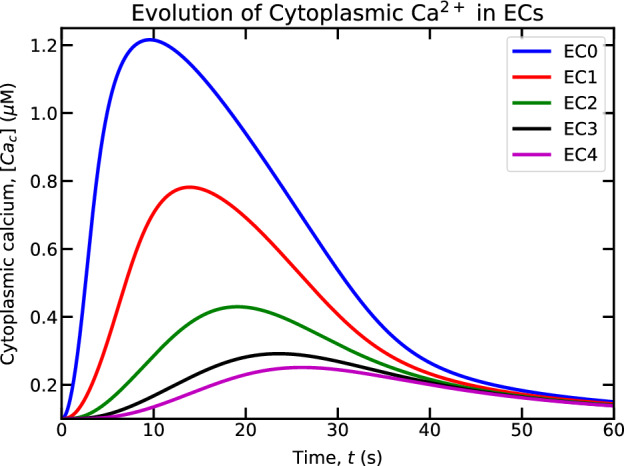
Evolution of cytoplasmic Ca$^{2+}$ concentration for the stimulated EC and also ECs on the right hand side of that EC. The stimulated EC is treated with 1 $\mu$M concentration of ATP.

Because of the symmetry of the problem, we present the evolution of cytoplasmic Ca$^{2+}$ (shown in Fig. 10) for ECs on the right hand of the stimulated EC (“EC0”) such as “EC1”, “EC2”, “EC3”, and “EC4”. As we move away from “EC0”, we find variations in cytoplasmic Ca$^{2+}$ peak magnitude as well as in peak time in the neighboring ECs. The increase in Ca$^{2+}$ concentration in ECs neighboring to “EC0” is due to the fact that IP$_3$ molecules diffuse from “EC0” to “EC1” and eventually to other ECs. As a consequence, the action of these molecules on IP$_3$R channels results in release of Ca$^{2+}$ from the ER. In Fig. 10, we find that the Ca$^{2+}$ peak in “EC1” occurs approximately 2 s after that in “EC0”. Therefore, in the average sense, the Ca$^{2+}$ signal moves at a speed of 5 $\mu$m/s. As we move further away from “EC1”, the speed of the Ca$^{2+}$ signal decays. This is due to the fact that the concentration of IP$_3$ decreases as it moves away from “EC0” because of passive diffusion of IP$_3$ molecules between ECs (Eq. (36)) and the degradation of its concentration in ECs (third term in Eq. (34)). Consequently, a decline in the cytoplasmic Ca$^{2+}$ peak concentration is observed (shown in Fig. 10).

In experiments^53–55,62^, it has been reported that the Ca$^{2+}$ wave can propagate at a speed of 10 $\mu$m/s or more, which compares well with the above order of magnitude. However, an important remark is in order. We have not observed a Ca$^{2+}$ wave front movement in our simulations, strictly speaking, in contrast to experiments^53–55,62^. It is likely that for wave front propagation to occur, an active generation of Ca$^{2+}$ or IP$_3$ would be required in the neighboring cells while Ca$^{2+}$ or IP$_3$ spreads from the stimulated cell through cell-cell junction^54,62,63^. This effect will be thoroughly investigated in the future in order to have a complete picture of wave propagation.

## Conclusions

In this study, we have successfully integrated the dynamic behavior of RBCs within a parabolic flow with the intricate dynamics of calcium ions (Ca$^{2+}$). While the primary function of RBCs is to facilitate gas exchange between lungs and tissues, they also play a crucial secondary role. RBCs release ATP as a signaling molecule in response to externally imposed flow. These ATP molecules act as messengers, transmitting signals from the external environment to the ECs, initiating the release of ubiquitous Ca$^{2+}$ ions. The presence of Ca$^{2+}$ ions plays a pivotal role in regulating numerous enzymatic functions. Notably, Ca$^{2+}$ governs the production of NO, a vasodilator crucial for maintaining vascular tone and overall cellular function. Consequently, this intricate system occurring in the vascular wall plays a vital role in both cellular regulation and vascular homeostasis.

To delve into the intricate relationship between RBC dynamics and biochemical signaling, we conducted a series of numerical simulations. By systematically varying parameters such as channel width (*Cn*), flow strength (*Ca*), and RBC concentrations (*Hct*) in a straight channel, we sought to uncover the underlying mechanisms. Employing the immersed boundary lattice Boltzmann method (IBLBM), we solved fluid-RBC interactions and ATP release mechanisms. Our investigation addressed a range of unanswered questions, including the impact of maximum ATP release levels on wall ATP concentration, the variation of ATP concentrations on vascular walls, the interplay between flow strength and confinement, and the potential existence of Ca$^{2+}$ wave propagation within a vascular network.

The main outcomes of the current study are summarized as follows: (1) Ectonucleotidase enzymes on the blood vessel walls play a critical role in preventing an abnormal rise in plasma ATP concentration and the activation of biochemical pathways in ECs. (2) There is no variation in steady ATP concentration along the flow direction, as well as minimal difference in its concentration between the bottom and top walls. (3) For a given channel length, hematocrit (*Hct*), and capillary number (*Ca*), the number of RBCs is higher in wider channels than in smaller channels with changes in *Cn*. Consequently, wider channels have a higher ATP concentration due to the increased total ATP release level from RBCs. (4) With an increase in *Ca* for a given *Cn*, changes in *Hct* lead to a monotonic increase in ATP concentration, resulting in corresponding monotonic rises in cytoplasmic Ca$^{2+}$ peak concentrations. This response is linear in dilute RBC concentrations; however, it becomes nonlinear in concentrated RBC concentrations due to the increase in cell-cell hydrodynamic interactions pushing RBCs towards the channel walls. This leads to an increase in ATP release due to higher membrane stress experienced by cells near the walls. (5) Similarly, keeping *Ca* constant, an increase in *Hct* leads to an increase in ATP concentration for a given *Cn*. The ATP concentration in wider channels is higher than that in smaller channels. This is due to the fact that a greater number of RBCs are flowing in wider channels compared to smaller channels, resulting in a higher total ATP release level from RBCs. The response of cytoplasmic Ca$^{2+}$ peak concentrations follows a similar trend to the ATP response, with larger ATP concentrations leading to higher cytoplasmic Ca$^{2+}$ peak concentrations. (6) The cytoplasmic Ca$^{2+}$ peak time is shorter with higher ATP concentration, indicating a faster response. The difference in relative peak times between different magnitudes of ATP concentrations at various regions of the channel leads to the propagation of Ca$^{2+}$ waves at a speed of 5 $\mu$m/s upstream and downstream of the channel. This is demonstrated by locally stimulating an EC within a confluent layer of ECs with ATP concentration, resulting in the release of Ca$^{2+}$ in neighboring ECs. This is mainly attributed to the diffusion of IP$_3$ molecules from a stimulated cell to neighboring cells, causing the release of sequestered Ca$^{2+}$ from the ER. The passive propagation of the Ca$^{2+}$ wave continues until IP$_3$ molecules successfully activate IP$_3$R channels present on the ER membrane. The propagation of the Ca$^{2+}$ wave upstream from a region in a vascular network helps coordinate cellular functions and control the flux of RBCs to fulfill the metabolic needs required for cellular functions.

While our study was conducted within a straight channel, it establishes a solid foundation for understanding RBC dynamics in the context of biochemical signaling under different (patho)physiological conditions. Our work can be extended to more complex vascular networks, where RBCs experience varying shear rates, local variations in RBC concentrations, ATP concentrations across different branches, and the propagation of Ca$^{2+}$ signals throughout upstream and downstream vessel branches. These expansions would significantly contribute to our understanding of localized blood flow regulation in real vasculature. In current study, we limited ourselves to 2D, which, at least, should provide qualitative results. this constitute an essential step in this broad area of research which is still in its nascent stage. This is also motivated by the limited availability of quantitative experimental data. For example, experiments regarding Ca$^{2+}$ signaling of endothelial cells (ECs) in the presence of blood flow are largely missing. The present study can be viewed as guide for future experiments before dealing with a full advanced 3D model.

Moreover, beyond ATP release, RBCs are also recognized as potential scavengers of NO, a vasodilator^64^. Therefore, a comprehensive investigation is required to unveil the intricate interplay between factors such as the cell-free layer (CFL), ATP concentration, RBC concentration, Ca$^{2+}$ concentration, and their combined effects on the bioavailability of NO within the vascular wall. Such endeavors would not only advance our scientific knowledge but also provide insights into the multifaceted mechanisms governing vascular health and function. Furthermore, incorporating membrane viscosity into the RBC membrane model influences the dynamics of RBCs, including deformation, suspension viscosity, and particle stress^48,49^. It would be interesting to explore the effect of membrane viscosity on ATP release from RBCs under various conditions and to quantitatively validate it with experimental results in future studies^2^.

## Data Availability

All outputs studied during current work are included in this published paper.
